# Preclinical Evaluation of [^18^F]LCATD as a PET Tracer to Study Drug-Drug Interactions Caused by Inhibition of Hepatic Transporters

**DOI:** 10.1155/2018/3064751

**Published:** 2018-07-30

**Authors:** Andrea Testa, Sergio Dall'Angelo, Marco Mingarelli, Andrea Augello, Lutz Schweiger, Andrew Welch, Charles S. Elmore, Dana Dawson, Pradeep Sharma, Matteo Zanda

**Affiliations:** ^1^Kosterlitz Centre for Therapeutics and John Mallard Scottish P.E.T. Centre, University of Aberdeen, Foresterhill, Aberdeen AB25 2ZD, UK; ^2^Early Chemical Development, Pharmaceutical Sciences, IMED Biotech Unit, AstraZeneca R&D, Pepparedsleden 1, 431 50 Mölndal, Sweden; ^3^Safety and ADME Translational Sciences, Drug Safety and Metabolism, IMED Biotech Unit, AstraZeneca R&D, Cambridge CB4 0WG, UK; ^4^C.N.R.–I.C.R.M., Via Mancinelli 7, 20131 Milan, Italy

## Abstract

The bile acid analogue **[** ^**18**^ **F]LCATD** (LithoCholic Acid Triazole Derivative) is transported in vitro by hepatic uptake transporters such as OATP1B1 and NTCP and efflux transporter BSEP. In this in vivo “proof of principle” study, we tested if **[** ^**18**^ **F]LCATD** may be used to evaluate drug-drug interactions (DDIs) caused by inhibition of liver transporters. Hepatic clearance of **[** ^**18**^ **F]LCATD** in rats was significantly modified upon coadministration of rifamycin SV or sodium fusidate, which are known to inhibit clinically relevant uptake transporters (OATP1B1, NTCP) and canalicular hepatic transporters (BSEP) in humans. Treatment with rifamycin SV (total dose 62.5 mg·Kg^−1^) reduced the maximum radioactivity of **[** ^**18**^ **F]LCATD** recorded in the liver from 14.2 ± 0.8% to 10.2 ± 0.9% and delayed *t*__max_ by 90 seconds relative to control rats. AUC_liver 0–5 min_, AUC_bile 0–10 min_ and hepatic uptake clearance CL_uptake,in vivo_ of rifamycin SV treated rats were significantly reduced, whereas AUC_liver 0–30 min_ was higher than in control rats. Administration of sodium fusidate (30 mg·Kg^−1^) inhibited the liver uptake of **[** ^**18**^ **F]LCATD**, although to a lesser extent, reducing the maximum radioactivity in the liver to 11.5 ± 0.3%. These preliminary results indicate that **[** ^**18**^ **F]LCATD** may be a good candidate for future applications as an investigational tracer to evaluate altered hepatobiliary excretion as a result of drug-induced inhibition of hepatic transporters.

## 1. Introduction

Hepatocytes perform most of the liver functions and are responsible for the detoxification of exogenous substances, including drug elimination through metabolism and/or biliary excretion processes. Drugs that cannot passively cross the lipid core of the hepatocyte membrane can be actively transported into hepatocytes by uptake transporter proteins. Within the hepatocyte, drugs may undergo metabolism and their metabolites may be transported by efflux transporters, either back into blood by basolateral efflux transporters or excreted into bile by canalicular transporters. Drugs and/or drug metabolites can competitively inhibit hepatic transporters, leading to pharmacokinetic interactions with coadministered drugs (or drug-drug-interactions, DDIs) that may result in serious adverse effects due to reduced drug clearance or intracellular accumulation of these drugs or their metabolites in hepatocytes [1, 2].

**Figure 1:**
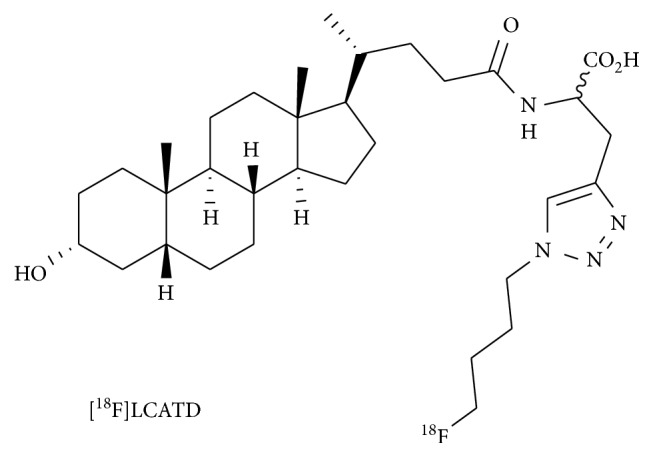
Chemical structure of **[** ^**18**^ **F]LCATD**.

With the aim to detect DDIs at an early stage of drug development and increase the understanding of hepatobiliary transport and its perturbation by action of clinically used drugs in vivo, considerable efforts have been dedicated to develop PET tracers for the study of hepatic transporters [3–5]. In this context, we previously described a lithocholic acid derivative, **[** ^**18**^ **F]LCATD** (Figure 1), which is a promising PET tracer to study the potential of clinically used drugs for hepatic transporter-mediated DDIs and hepatotoxicity [6]. In vitro studies confirmed that **[** ^**18**^ **F]LCATD** appears to be a substrate of membrane uptake transporters OATP1B1 and NTCP (while being a low affinity substrate of OATP1B3) and is excreted into the bile via the efflux transporter BSEP. **[** ^**18**^ **F]LCATD** is characterized by low passive permeability and, as demonstrated by a preliminary imaging experiment in rats, it rapidly accumulated into the liver to be then excreted exclusively into the bile [6].

Here, we present the results of preclinical PET imaging studies performed to assess the potential of **[** ^**18**^ **F]LCATD** to image in vivo the effects of rifamycin SV and sodium fusidate on hepatic uptake and biliary excretion when coadministered via bolus injection. In vitro studies confirmed earlier that **[** ^**18**^ **F]LCATD** acts as a probe for endogenous bile acid transport and can be used for preclinical evaluation of drug interference at the level of hepatic bile acid transporters function. Although in vitro assays were performed in cells expressing human OATP1B1, we chose the rat model for our investigations as the human and the rat orthologs of OATP1B1 exhibit very similar transport characteristics. Furthermore, rats have been used extensively as animal models for studies on liver transporters [3, 4, 7] and for the evaluation of transporters targeting PET tracers, such as [^11^C]dehydropravastatin [8], [^11^C]rosuvastatin [7], [^11^C-]telmisartan [9], [^11^C]*N*-acetyl leukotriene E4 [10],0 and (15*R*)- 16-*m*-[^11^C]tolyl-17,18,19,20-tetranorisocarbacyclin methyl ester [11].

## 2. Materials and Methods

### 2.1. Chemicals and Radiotracer

For preclinical PET studies, **[** ^**18**^ **F]LCATD** was synthesised as reported previously [6] (see the Supplementary Materials for further details) and formulated in 10% ethanol in PBS. Radiochemical purity was >99% and specific activity >20 GBq·*µ*mol^−1^. Rifamycin SV was purchased from Sigma Aldrich (UK) and dissolved in 10% ethanol in saline solution (20 mg·mL^−1^). Sodium fusidate was purchased from Sigma-Aldrich (UK) and was formulated in saline solution (20 mg·mL^−1^).

### 2.2. Animals

Female Sprague-Dawley (SD) rats (200–250 g) were purchased from Harlan (UK). The animals were provided with standard food and water at libitum in a temperature and light-controlled environment. All animal experiments were performed according to the Aberdeen University's Code of Practice on the Use of Animals in Research as well as the legal requirements of the Animals (scientific procedures) Act 1986 and Home Office Code of Practice guidance. At the end of the imaging experiments, animals were culled by cardiac puncture (schedule 1 technique, exsanguination) while under anaesthesia, and then a terminal blood sample, liver, kidney, and heart were collected.

### 2.3. PET/CT Imaging

PET scans were performed with a preclinical PET/CT SEDECAL ARGUS scanner (SEDECAL, Spain) housed in a temperature-controlled suite. The scanner has two 11.8 cm diameter rings of photoswitch detectors coupled to position-sensitive photomultiplier tubes, giving a 4.8 cm axial and 7.5 cm transaxial field of view (FOV). All animals were cannulated (Venisystem Butterfly, Abbott Ireland) in the tail vein for tracer injection under general anaesthesia with isoflurane (IsoFlo 100% w/w, Abbott Laboratories Ltd) 2.5% (2 L·min^−1^ oxygen flow). Animals were divided into three groups: control group (administered with **[** ^**18**^ **F]LCATD** only, 3 animals), rifamycin SV-treated group (3 animals) and fusidate-treated group (3 animals). All the animals were scanned in supine position. At the start of the scan, **[** ^**18**^ **F]LCATD** (5–15 MBq per body) was injected as a single bolus into the tail vein. Administered radioactivity was measured as the net counts in the syringe before and after the injection with an ionization chamber (Capintec CRC-15PET; Ramsey, New Jersey). The mass amount of **[** ^**18**^ **F]LCATD** administered per animal was estimated to be between 0.2 and 1 nmol. For inhibition experiments, rifamycin SV was administrated 45 minutes before the start of the scan at a dose of 50mg·Kg^−1^ (intraperitoneally) and at the time of the tracer injection at a dose of 12.5 mg·Kg^−1^ (intravenously). For the in vivo assessment of the inhibitory effect of sodium fusidate on the hepatobiliary transport of the tracer, the drug was administrated at a dose of 20 mg·Kg^−1^ (intraperitoneally) 30 minutes before the scan and at a dose of 10 mg·Kg^−1^ (intravenously) after 15 minutes from the tracer injection. Emission scans in 3D list-mode were acquired for 30 minutes (with a FOV encompassing part of the thorax and the intestine) followed by a 5 minutes CT scan. PET acquisitions were obtained with an energy window set to 250–700 keV and corrections for dead-time, activity decay, attenuation, random, and scatter counts were applied (using the manufacturer' software). The dynamic images were obtained by sorting the list mode data into the following time frames: 6 × 10 s, 8 × 30 s, 10 × 60 s, and 3 × 300 s, and images were reconstructed with a FORE/2D-OSEM reconstruction algorithm (provided by the manufacturer). Sampling at shorter intervals during early time-points enabled us to detect rapid increments of tracer uptake in the liver and intestine. Longer time frames at later time-points from the injection time reduced noise in the images and showed the amounts of uptake characteristic for the specific PET tracer. A 5-minute PET scan of the injection site at the end of the CT was obtained to ensure successful intravenous injection. Cross calibration between the ionization chamber and the PET scanner was carried out by using a cylindrical phantom made of Perspex with internal diameter of 43 mm and length of 37 mm. 10 to 20 minute PET scans of the phantom filled with an average of about 4.20 MBq of **[** ^**18**^ **F]LCATD** in 60 g of water were acquired (average dead-time value 4%).

The registered PET and CT images were transferred from the scanner to a processing workstation, and subsequent analysis was carried out using Pmod (Pmod Technologies, CH) version 3.2. Regions of interest (ROIs) corresponding to abdominal aorta, liver, and gastrointestinal tract were defined via a combination of manual and threshold methods (Supplementary Figure 1 and movie) using PMOD software (PMOD Technologies). The stomach was included in the gastrointestinal ROI to include the amount of bile that might have refluxed from the duodenum into the stomach.

### 2.4. Postmortem Biodistribution of the Tracer

Samples of the liver, heart, and kidney were collected at the end of the PET-CT scan (after euthanasia) and homogenised with methanol (80% in water, 5 mL per gram of biological sample) by means of a homogenizer. Samples were stored in preweighed plastic vials. Terminal blood was collected by cardiac puncture just before euthanasia. The activity of the homogenates and blood samples was measured by means of a precalibrated gamma counter as soon as possible after the PET experiment.

### 2.5. Postmortem Metabolites Analysis

Analysis of metabolites in the blood, liver, and bile (extracted from the intestine) was performed via radio-HPLC. Blood samples, collected by cardiac puncture at the end of the PET-CT scan, were treated with an equal volume of acetonitrile, centrifuged for 1 minute at 1000 rpm and the supernatant was injected in radio-HPLC (Shimadzu Prominence HPLC system equipped with a PDA UV detector and HERM LB500 activity detector and Phenomenex Luna C18 column, 5 *μ*m, 100 Å, 250 × 4.6 mm, injection volume 20 *µ*L, PBS/CH_3_CN 60 : 40, isocratic elution, flow: 1 mL·min^−1^). Samples of the liver and duodenum were collected at the end of the PET-CT scan (after euthanasia) homogenated with methanol (80% in water, 5 mL per gram of biological sample) by means of a homogenizer and centrifuged for 1 minute at 1000 rpm and the supernatant was injected in radio-HPLC.

### 2.6. Data Analysis

Uptake hepatic clearance CL_uptake,in vivo_ was calculated by integration plots obtained applying the Patlak method in Pmod (Pmod Technologies, CH) version 3.2, limiting the linear fit to the uptake phase (0–3 min) [3, 12].

Intrinsic biliary clearance of the radiotracer CL_int,bile_ was determined as proposed by Takashima et al. [11] by quantification of the radiotracer in the liver and the intestine (as amount of radiotracer excreted into the bile).

An ANOVA single-factor analysis (Microsoft Excel 2013) was used to check whether there were statistically significant differences between AUC of blood, liver, and bile of three groups (control, rifamycin, and fusidate treated rats). For AUC_liver_, there was a statistically significant difference between groups, *p*=0.01; for AUC_blood_, there were no statistically significant differences between group means, *p*=0.7; for AUC_bile_, there was a statistically significant difference between groups, *p*=0.03.

Two-tailed Student's *t*-test (Microsoft Excel 2013) with equal variances was used to determine level of significance of various PK parameters between control and drug-treated animals at various time periods.

## 3. Results

### 3.1. Concentration Profiles of **[** ^**18**^ **F]LCATD** in Arterial Blood

The PET tracer concentration in arterial blood was derived from the PET images by observing the activity of a clearly visible portion of the abdominal aorta (Figure S4, Supplementary Materials). The image-derived arterial concentration of the PET tracer is likely to be affected by errors due to the small dimensions of the blood vessel in comparison to the scanner's spatial resolution. Indeed, contamination due to spillover from other vicinal organs and partial volume effects are expected to increase and reduce respectively (by unknown amounts) the actual activity of the arterial blood. However, most of these effects should be similar among different animals, allowing the comparison between treated and untreated groups.

**Figure 2:**
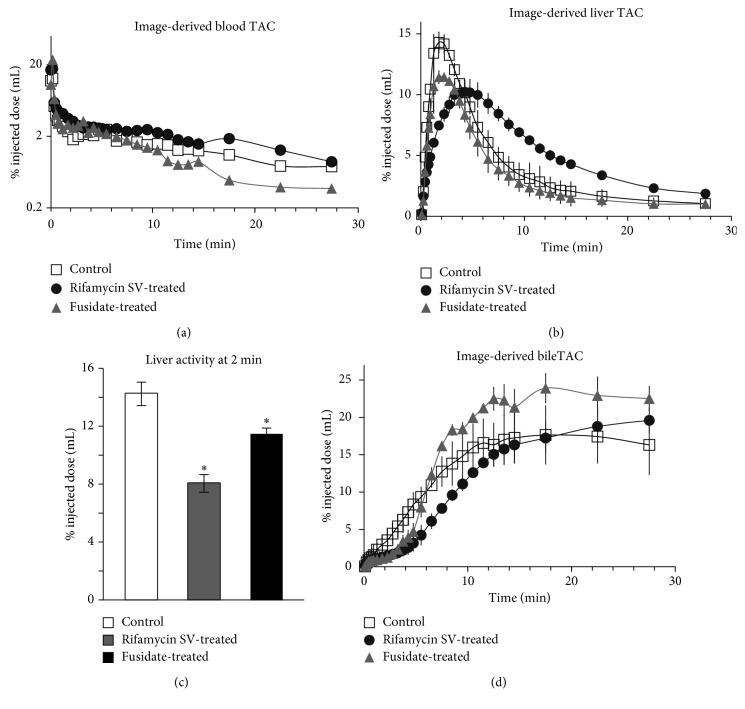
(a) Time-activity profiles obtained from the images of the abdominal aorta of control, rifamycin SV-treated, and fusidate-treated rats. No statistical significance of rifamycin-SV and fusidate-treated rats compared to the control was found at any point (error bars omitted for clarity). (b) Time-activity profiles obtained from the images of the liver of control, rifamycin SV-treated, and fusidate-treated rats as mean ±1 s.d., *n*=3. (c) Time-activity profiles for the bile, obtained from the images of the gastrointestinal region of control, rifamycin SV-treated, and fusidate-treated rats as mean ±1 s.d., *n*=3. (d) Activity in the liver of control, rifamycin SV-treated, and fusidate-treated rats at 2 minutes postinjection determined noninvasively via PET imaging, as mean ±1 s.d., *n*=3. Statistical significance of rifamycin-SV and fusidate-treated rats compared to the control was assessed by two-tailed *t*-test assuming equal variances, ^*∗*^ *p* < 0.05.

The blood time-activity curves (TAC) obtained for control, rifamycin SV-treated and fusidate-treated animals are reported in Figure 2(a). The blood concentration of the tracer in rifamycin SV-treated animals was higher than the PET tracer concentration measured in control rats, but the differences between the relative TAC curves were not significant (for clarity the standard deviation bars are not shown in Figure 2(a)). The blood concentration of the tracer in fusidate-treated animals was instead lower than the PET tracer concentration measured in the blood of control rats, but again the differences between the relative TACs were not significant.

### 3.2. Liver Uptake and Biliary Efflux of **[** ^**18**^ **F]LCATD**

**Figure 3:**
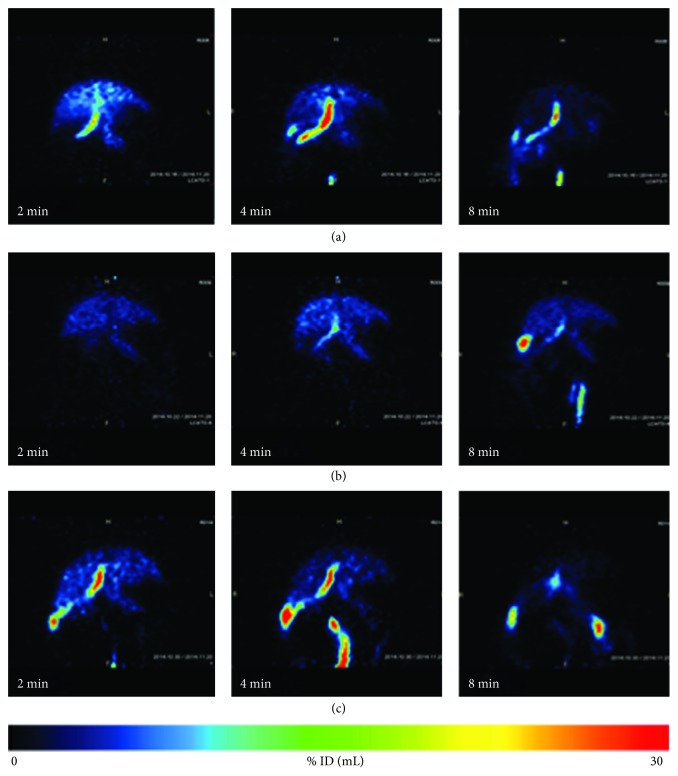
Maximum intensity coronal projection of livers of a representative control (a), rifamycin SV-treated (b), and fusidate-treated (c) rat at 2, 4, and 8 minutes postinjection.

Coronal representative PET images of the liver of control, rifamycin SV-treated, and fusidate-treated rats are reported in Figure 3. Reduced exposure to radioactivity in the liver of rifamycin SV-treated rats as well as delayed biliary excretion of the tracer can be clearly observed, whereas no obvious differences can be noticed between control and fusidate-treated rats.

TAC for the liver of control, rifamycin-treated, and fusidate-treated rats are shown in Figure 2(b). In control rats, a maximum of 14.2 ± 0.8% of the injected dose was found in the liver 150 seconds postinjection. Rapid washout was observed, and after 30 minutes, the residual activity measured in the liver was 1.04 ± 0.17% of the injected dose. In rifamycin SV-treated rats, the maximum activity recorded in the liver was 10.2 ± 0.9% of the injected dose 225 seconds postinjection. The washout phase was clearly delayed, and after 30 minutes, the activity in the liver was 1.87 ± 0.13% of the injected dose. Treatment with sodium fusidate reduced the maximum activity registered in the liver to 11.5 ± 0.3% of the injected dose 135 seconds after tracer injection. The TAC profile was similar to the one obtained for control rats, with a fast washout that reduced the activity in the liver to 1.02 ± 0.02% of the injected dose after 30 minutes (Table 1). Quantitative PET analysis showed that the activity found in the liver 2 minutes after tracer injection in control, rifamycin SV-treated, and fusidate-treated rats (Figure 2(d)) is significantly different among the groups (*p* < 0.05). At this time-point, 14.2 ± 0.8% of the injected activity was found in the liver of control rats, while for the rifamycin SV-treated and sodium fusidate-treated rats, the liver activity was reduced to 8.0 ± 0.6% and 11.4 ± 0.4% respectively.

The area under the curve for the liver from 0 to 5 minutes (AUC_liver 0–5 min_) for rifamycin SV-treated rats was significantly lower than that of control rats, and the same observation can be made for the AUC_liver 0–5 min_ of fusidate-treated rats, while no statistically significant difference was found between the liver AUC_liver 0–30 min_ of the three animal groups (Table 1).

In order to calculate the liver uptake clearance CL_uptake,in vivo_, integration plots were obtained applying the Patlak method in Pmod, limiting the linear fit to the uptake phase [3]. In this study, the blood time-concentration input function was derived from **[** ^**18**^ **F]LCATD**'s activity in the abdominal aorta; therefore, CL_uptake,in vivo_ values so calculated cannot be compared with those obtained for different tracers from literature, whose blood concentrations were determined by measuring the activity of blood samples [3]. The CL_uptake,in vivo_ of rifamycin SV-treated and fusidate-treated rats were significantly lower than the CL_uptake,in vivo_ of control rats (Table 1).

As shown by the bile TACs reported in Figure 2(c), the radiotracer's biliary excretion was delayed in rifamycin SV-treated and fusidate-treated rats, while for control rats, a straight line was observed between 0 and 200 seconds and a plateau was observed in the case of the drug-treated rats. The area under the curve for the bile from 0 to 10 minutes (AUC_bile 0–10 min_) of rifamycin SV-treated rats was significantly lower than the one obtained for control rats, whereas no difference was found among control and fusidate-treated rats. Differences in the intrinsic biliary clearance of the radiotracer CL_int,bile_ [3] were found not to be statistically significant between control and rifamycin SV-treated rats, whereas sodium fusidate treatment significantly increased this value (Table 1).

### 3.3. Postmortem Biodistribution of the Tracer

**Figure 4:**
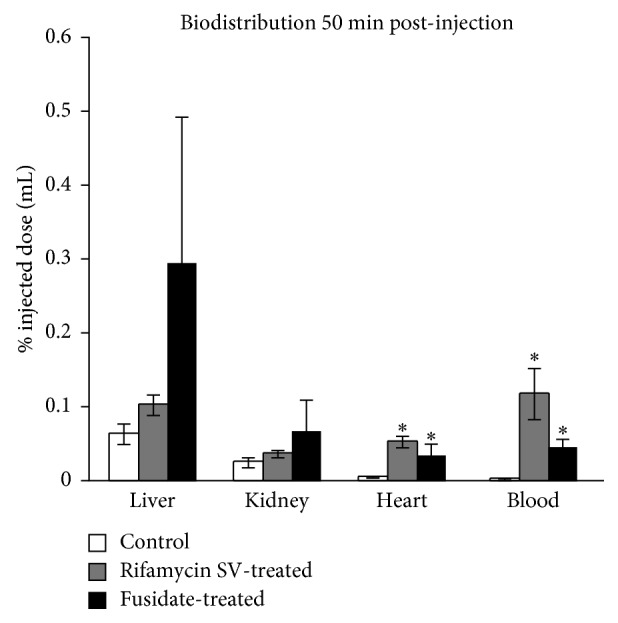
Activity in the liver, kidney, heart, and blood of control, rifamycin SV-treated, and fusidate-treated rats at 50 minutes postinjection as mean ±1 s.d., *n*=3. Statistical significance of rifamycin-SV and fusidate-treated rats compared to the control was assessed by two-tailed *t*-test assuming equal variances, ^*∗*^ *p* < 0.05.

Activity found in the liver of control, rifamycin SV-treated, and fusidate-treated animals 50 minutes postinjection was extremely low (Figure 4), as expected from the fast clearance previously observed for the tracer, and no statistically significant differences were found among the three experimental groups. Neither rifamycin SV nor sodium fusidate significantly affected the biodistribution of the tracer in kidneys 50 minutes postinjection, which was extremely low in all the animal groups. In fact, the renal clearance of **[** ^**18**^ **F]LCATD** appears negligible if compared with the biliary clearance, as kidneys were not visible in the PET scan images, despite being in the field of view. Both rifamycin SV and sodium fusidate significantly increased the activity in the terminal blood sample, and higher levels of radioactivity were found in the heart of treated animals. This is consistent with a reduced liver uptake and excretion, which are both expected to increase the PET tracer's blood concentration.

## 4. Discussion

The PET tracer **[** ^**18**^ **F]LCATD** is a bile acid analogue, closely related to lithocholic acid, that can be used as a probe to evaluate the inhibition of liver transporters. In vitro cell uptake and membrane vesicle efflux assays using [^3^H]LCATD demonstrated that this amphiphilic molecule can hardly penetrate the cell membrane by passive diffusion, whereas it is quickly taken up by cells expressing the OATP1B1, NTCP transporters, and effluxed by means of BSEP [6]. Involvement of other uptake transporters (e.g., OATP2B1, OATP1A2, OAT2, and OAT7) and apical transporters (such as MATE1, MRP2, BCRP, and MDR1/3) could not be excluded. A preliminary in vivo PET study showed that, after intravenous injection, **[** ^**18**^ **F]LCATD** selectively accumulates in the liver [6], the sole organ in which the murine homologs of such transporters are significantly expressed.

Being **[** ^**18**^ **F]LCATD** a substrate of the pharmacologically relevant hepatic transporter OATP1B1, as well as of transporters that are normally involved in the uptake and clearance of bile acids (such as NTCP and BSEP), if the coadministration of a drug affects the biodistribution of **[** ^**18**^ **F]LCATD**, then there are high chances that the drug could cause DDIs or possibly drug induced hepatoxicity. As a preclinical proof of concept, this study was focused on the effects of the coadministration of two drugs known to inhibit hepatic transporters, for example, rifamycin SV and sodium fusidate, on the hepatobiliary distribution of **[** ^**18**^ **F]LCATD**. Rifamycin SV has been widely used in vitro as a pharmacologically relevant transporter inhibitor [13–16], and we have previously demonstrated that it could inhibit the uptake of tritium-labelled LCATD in an OATP1B1 cell-based assay [6]. We therefore anticipated that rifamycin SV could represent a benchmark inhibitor for this imaging study. On the other hand, sodium fusidate was selected as transporters inhibitor for this study because of its involvement in clinically relevant DDIs. Fusidate-induced hepatic transporters inhibition has indeed been proposed as a possible cause of myopathy including rhabdomyolysis upon coadministration with statins [17, 18].

To date, a small number of bile acids derivatives labelled with gamma and positron emitting radioisotopes have been developed with the aim of studying hepatobiliary and intestinal transport by means of SPECT and PET imaging. [^75^Se]25-homotaurocholic acid (SeHCAT) [19] was the first gamma-emitting bile acid derivative, developed in 1979. [^11^C]Cholylsarcosine, an analogue of the bile acid cholylglycine firstly reported in 2012 [20], has been shown to be transported by bile acid transporters in pigs [21] and proved to be a useful PET tracer to study biliary secretion in healthy humans and patients with cholestasis [22]. Soon after our first report of **[** ^**18**^ **F]LCATD** as the first [^18^F]fluorine-labelled bile acid [6], another derivative of cholic acid, 3*β*-[^18^F]fluorocholic acid ([^18^F]FCA) was reported by De Lombaerde et al. [23] as a promising probe to monitor altered hepatobilary transport in vivo during drug development. Compared to other PET tracers developed to study liver transporters which are transported quite selectively by the OATPs and MRP2 and BCRP [3, 4] bile acid-based tracers such as **[** ^**18**^ **F]LCATD**, [^18^F]FCA and [^11^C]Cholylsarcosine are generally handled by the same transporters involved in uptake and efflux of endogenous bile acids (NTCP, OATP1B1, OATP1B3, BSEP, and MRP2) [24–26], making them the best candidates to study physiology and pathophysiology of the hepatic bile acid transport in vivo [22, 23, 27].

As observed for the other bile acid-based [^18^F]fluorine-labelled PET tracer [^18^F]FCA [23], **[** ^**18**^ **F]LCATD** reached a peak concentration in the liver in a very short time (*t*__max_ = 135 seconds) in untreated animals.

Treatment with rifamycin SV reduced the maximum activity recorded in the liver from 14.2 ± 0.8% to 10.2 ± 1.0% and delayed *t*__max_ by 90 seconds, indicating an inhibitory effect of the drug on the transporters involved in the uptake of **[** ^**18**^ **F]LCATD**. In rifamycin SV-treated rats the AUC_liver 0–5 min_ and the hepatic uptake clearance CL_uptake,in vivo_ were also significantly reduced as a consequence of the effect of the drug on uptake transporters (Table 1). If compared with the TAC obtained for control rats, rifamycin SV-treated rats showed a less steep exponential decay during the phase representing the excretion of the tracer from the hepatocytes. Consequently, the AUC_liver 0–30 min_ of rifamycin SV-treated rats was higher than the AUC_liver 0–30 min_ of control rats, while the AUC_bile 0–10 min_ of rifamycin SV-treated rats was significantly lower than the AUC_bile 0–10 min_ of control rats, suggesting an inhibitory effect on efflux transporters. It is worth mentioning that although AUC_liver 0–5 min_ (which describes the accumulation of the probe in the liver) and CL_uptake,in vivo_ (a kinetic parameter describing the transport of probe substrate) are closely related, it is difficult to correlate them based on available information. Since uptake transporters are inhibited by rifamycin SV, it is expected that this will increase plasma/blood concentrations of the PET tracer, while decreasing accumulation in liver reflected in a decrease of AUC_liver_. This is confirmed by the *K*_p liver_ (tissue/plasma ratio) calculated for the three groups. Taken together, this data are in accordance with the inhibitory activity of rifamycin SV previously observed on both uptake and canalicular transporters. In fact, rifamycin SV has been shown to inhibit OATP1B1, OATP1B3, NTCP [16, 28], and the ATP-dependent bile salt export pump (BSEP) [29, 30]. Inhibition of OATP1B1 and BSEP is expected to occur when the serum-free concentration of rifamycin SV is higher than its IC_50_ for these transporters. The reported IC_50_ values of rifamycin SV for OATP1B1 and BSEP are, respectively, 0.2–0.4 *µ*M [6, 16] and 6.3 *µ*M [31], while a *K*_*i*_ value >63 *µ*M for NTCP has been reported [13]. The administered dose of rifamycin SV was 62.5 mg·Kg^−1^, and taking into account the IC_50_ values indicated above, it is possible that rifamycin SV plasma concentrations would be enough to inhibit OATP1B1 and BSEP, whereas inhibition of NTCP may only be partial. These observations would be consistent with the results of the PET imaging experiments. The administration route of rifamycin SV and dose used in our protocol were based on a previous study in which the structurally related inhibitor rifampicin was used as hepatic transporters inhibitor in mice [32]. Inhibition of hepatic transport was seen at a dose of 37.5 mg·Kg^−1^ intraperitoneally and 9.37 mg·Kg^−1^ intravenously [32]. This is in line with the observed inhibition of **[** ^**18**^ **F]LCATD** transport at the dose used in our work (50 mg·Kg^−1^ ip and 12.5 mg·Kg^−1^ iv).

Postmortem analysis (50 minutes postinjection) of the residual activity in liver, kidney, heart, and blood was also consistent with the in vivo imaging results. In fact, a very low residual tracer concentration was found in the liver in line with the fast biliary clearance observed by PET imaging. Very low activity was found in the kidneys (<0.1% of the injected dose, comparable to other blood perfused organs like the heart) and significant differences (*p* < 0.05) were found between the tracer concentrations in the blood of control and rifamycin-treated rats, as a result of the drug-induced impaired hepatic clearance.

Although we could not detect **[** ^**18**^ **F]LCATD** or any radiometabolite in blood and liver extracts, radio-HPLC analysis of intestine extracts of two animals from the control group showed a twin peak matching **[** ^**18**^ **F]LCATD** that accounted for over 90% of the activity (see Figure S1, Supplementary Materials), suggesting that the tracer is at least 90% metabolically stable 50 min postinjection.

Coadministration of sodium fusidate (30 mg·Kg^−1^) decreased the liver uptake of **[** ^**18**^ **F]LCATD**, although to a lesser extent compared to rifamycin SV: the maximum activity found in the liver was reduced from 14.2 ± 0.8% to 11.4 ± 0.3% but *t*__max_ was not delayed in this case. The lower AUC_liver 0–5 min_ and AUC_liver 0–30 min_ of fusidate-treated rats relative to control rats (although the latter was not significant) indicated that the hepatocytes had been exposed to lower amounts of PET tracer, as a likely result of the uptake inhibition. Interestingly, the AUC_bile 0–10 min_ of fusidate-treated rats was higher than the AUC_bile 0–10 min_ of control rats, even though the difference was not statistically significant. The CL_int,bile_ was also higher than that of control rats. Finally, significant differences were found between the tracer's concentrations in the terminal blood sample of control and fusidate-treated rats. All these results suggest that at the administrated dose, sodium fusidate inhibits the liver uptake of the tracer but not the canalicular efflux, which instead seems to be stimulated by the presence of the drug. This might be explained by considering that, in previous studies [33], fusidic acid (0.3–300 *µ*M) was shown to stimulate the MRP2 transporter (canalicular efflux) in vitro, as indicated by the increased efflux of LTC4 (leucotriene C4) using MRP2 transfected membrane vesicles. Many bile acid salts are known to be transported by MRP2 [34] and, being **[** ^**18**^ **F]LCATD** a bile acid analogue, it is possible that its MRP2-mediated excretion might have been stimulated by fusidic acid administration. However, for the scope of this study, we did not assess the involvement of MRP2 in the canalicular efflux of **[** ^**18**^ **F]LCATD**, and we recognise that future work should explore this aspect in greater detail.

The effect of sodium fusidate on the biliary transport has been previously studied in vivo in rats, and an overall reduced biliary clearance of [^3^H]-cholyltaurine was observed after administration of a 54 mg·Kg^−1^ dose [35]. However, the authors could not clarify if the effect was due to a reduced liver uptake of cholyltaurine or caused by a reduced canalicular efflux (thus increasing the hepatocyte exposure to the drug), or both. In a more recent study, sodium fusidate was shown to inhibit the hepatic uptake of rosuvastatin, via inhibition of OATP1B1 and OATP1B3, when orally administered in rats at a dose of 250 mg·Kg^−1^ [17].

In our study, the administered dose of sodium fusidate was 30 mg·Kg^−1^, which is comparable to the dose previously used by Bode (54 mg·Kg^−1^ ip) [35] to study the effect of fusidate on cholyltaurine transport in the rat liver. Although the actual plasma concentration could not be measured, the administered dose of fusidate may be enough to inhibit OATP1B1, BSEP, and NTCP. For these transporters, IC_50_ values of sodium fusidate have been reported to be in the micromolar range: 1.6 to 35 *µ*M for OATP1B1 [17, 18, 33], 3.8–11.5 *μ*M for BSEP [31, 33], and 44 *μ*M for NTCP [33]. The higher CL_int,bile_ observed for fusidate-treated rats could be explained as the net effect of fusidic acid on BSEP inhibition and MRP2 stimulation. The significant differences observed for fusidate-treated rats on AUC_liver 0–5 min_, on the radioactivity in the liver at 2 minutes postinjection and on CL_int,bile_ corroborate the hypothesis that **[** ^**18**^ **F]LCATD**, can be used as a tracer to identify perturbation of hepatic uptake and efflux transporters in vivo. However, in the case of fusidic acid, further studies with transporter knock-out rodent models may be necessary to unequivocally delineate the net uptake and efflux inhibition and the stimulation properties. Moreover, such knock-out rodent models may help to understand the role of other efflux transporters such as MATE1, BCRP, MRP2, and PgP in addition to BSEP to the canalicular efflux of **[** ^**18**^ **F]LCATD**.

We can speculate that, if the objective of the experiment is to determine whether a drug has potential for DDI, continuous blood infusion of the investigational drug and/or dose escalation may provide more conclusive information. However, limitations due to altered tracer metabolism and transporters expression induced by the administration of the investigational drug, as well as reduced blood flow due to anaesthetic procedures may need to be considered.

## 5. Conclusion

In conclusion, we have shown that hepatic clearance of the PET tracer **[** ^**18**^ **F]LCATD** (which had been previously characterized in vitro) (6)] is significantly modified in vivo, in preclinical models (rat), by the coadministration of rifamycin SV and sodium fusidate, which are known to cause the inhibition of clinically relevant uptake and canalicular hepatic transporters.

Since **[** ^**18**^ **F]LCATD** can be easily prepared in a standard fully automated radiosynthesis module [6] in any PET radiochemistry laboratory (even in a non-PET-specialised industrial environment) from commercially available aqueous [^18^F]fluoride solutions, we anticipate that this tracer could be advantageously used in the safety evaluation of investigational drugs early in the drug development phase. Moreover, the use of bile acid analogues such as **[** ^**18**^ **F]LCATD**, which are transported by bile acid transporters NTCP and BSEP, could be tested as diagnostic agents, in alternative to the very short lived [^11^C]cholylsarcosine, for imaging pathophysiological hepatic conditions in patients.

## Figures and Tables

**Table 1:** Kinetic parameters for **[** ^**18**^ **F]LCATD** as mean ±1 s.d., *n*=3.

Group	*t*__max_ (s)	C_max,liver_ (% ID·mL^−1^)	AUC_liver 0–5 min_ (% ID·min·mL^−1^)	*K_p_*_liver 0−5 min_	AUC_liver 0–30 min_ (% ID·min·mL^−1^)	AUC_bile 0–10 min_ (% ID·min·mL^−1^)	CL_uptake,in vivo_ (mL·min^−1^·g^−1^)	CL_int,bile_ (mL·min^−1^·g^−1^)
Control	135	14.3 ± 0.9	58 ± 1	3.64 ± 0.09	110 ± 15	89 ± 1	2.09 ± 0.27	0.17 ± 0.03
Rifamycin SV-treated	225	10.2 ± 0.9^*∗*^	44 ± 4^*∗*^	2.07 ± 0.17	139 ± 9	53 ± 3^*∗*^	0.71 ± 0.05^*∗*^	0.12 ± 0.03
Fusidate-treated	135	11.5 ± 0.3^*∗*^	48 ± 0.3^*∗*^	2.451 ± 0.016	89 ± 11	93 ± 19	1.40 ± 0.13^*∗*^	0.36 ± 0.03^*∗*^

Statistical significance of rifamycin-SV and fusidate-treated rats compared to the control was assessed by two-tailed *t*-test assuming equal variances, ^*∗*^ *p* < 0.05. *t*_max_: time to maximum peak concentration; C_max,liver_: maximum measured activity in the liver; AUC_liver 0–5 min_: area under the curve for the liver from 0 to 5 minutes postinjection; *K*_p liver 0–5 min_: apparent liver-to-blood AUC_0–5 min_ ratio; AUC_liver 0–30 min_: area under the curve for the liver from 0 to 30 minutes postinjection; AUC_bile0–10 min_: area under the curve for the bile (as radioactivity measured in the gastrointestinal tract) from 0 to 10 minutes postinjection; CL_uptake,in vivo_:in vivo uptake clearance; CL_int,bile_: intrinsic biliary clearance of the radiotracer.

## Data Availability

The data used to support the findings of this study are available from the corresponding author upon request.

## Supplementary Materials

Supplementary Materials The supporting information includes radio-HPLC analysis of bile extracts (Figure S1); the experimentalprotocol for the radiosynthesis of **[** ^18^ **F]LCATD**,module configuration (.doc file) (Figure S2); HPLC coinjectionwith LCATD (Figure S3); and visualisation of ROIs (Figure S4). Rotating ROIs are visualised in the movie file Video_ROIs.wmv.

## References

[1] Houfu L., Jasminder S. (2016). Role of hepatic drug transporters in drug development. *Journal of Clinical Pharmacology*.

[2] Patel M., Taskar K. S., Zamek-Gliszczynski M. J. (2016). Importance of hepatic transporters in clinical disposition of drugs and their metabolites. *Journal of Clinical Pharmacology*.

[3] Testa A., Zanda M., Elmore C. S., Sharma P. (2015). PET tracers to study clinically relevant hepatic transporters. *Molecular Pharmaceutics*.

[4] Langer O. (2016). Use of PET imaging to evaluate transporter-mediated drug-drug interactions. *Journal of Clinical Pharmacology*.

[5] Mann A., Han H., Eyal S. (2016). Imaging transporters: transforming diagnostic and therapeutic development. *Clinical Pharmacology & Therapeutics*.

[6] Testa A., Dall’Angelo S., Mingarelli M. (2017). Design, synthesis, in vitro characterization and preliminary imaging studies on fluorinated bile acid derivatives as PET tracers to study hepatic transporters. *Bioorganic & Medicinal Chemistry*.

[7] He J., Yu Y., Prasad B. (2014). PET imaging of oatp-mediated hepatobiliary transport of [(11)C] rosuvastatin in the rat. *Molecular Pharmaceutics*.

[8] Shingaki T., Takashima T., Ijuin R. (2013). Evaluation of Oatp and Mrp2 activities in hepatobiliary excretion using newly developed positron emission tomography tracer [11C]dehydropravastatin in rats. *Journal of Pharmacology and Experimental Therapeutics*.

[9] Takashima T., Hashizume Y., Katayama Y. (2011). The involvement of organic anion transporting polypeptide in the hepatic uptake of telmisartan in rats: PET studies with [(1)(1)C]telmisartan. *Molecular Pharmaceutics*.

[10] Hagmann W., Denzlinger C., Rapp S., Weckbecker G., Keppler D. (1986). Identification of the major endogenous leukotriene metabolite in the bile of rats as N-acetyl leukotriene E4. *Prostaglandins*.

[11] Takashima T., Nagata H., Nakae T. (2010). Positron emission tomography studies using (15R)-16-m-[11C]tolyl-17,18,19,20-tetranorisocarbacyclin methyl ester for the evaluation of hepatobiliary transport. *Journal of Pharmacology and Experimental Therapeutics*.

[12] Patlak C. S., Blasberg R. G. (1985). Graphical evaluation of blood-to-brain transfer constants from multiple-time uptake data. Generalizations. *Journal of Cerebral Blood Flow & Metabolism*.

[13] Fattinger K., Cattori V., Hagenbuch B., Meier P. J., Stieger B. (2000). Rifamycin SV and rifampicin exhibit differential inhibition of the hepatic rat organic anion transporting polypeptides, Oatp1 and Oatp2. *Hepatology*.

[14] Vavricka S. R., Van Montfoort J., Ha H. R., Meier P. J., Fattinger K. (2002). Interactions of rifamycin SV and rifampicin with organic anion uptake systems of human liver. *Hepatology*.

[15] Sharma P., Butters C. J., Smith V., Elsby R., Surry D. (2012). Prediction of the in vivo OATP1B1-mediated drug-drug interaction potential of an investigational drug against a range of statins. *European Journal of Pharmaceutical Sciences*.

[16] Sharma P., Holmes V. E., Elsby R., Lambert C., Surry D. (2010). Validation of cell-based OATP1B1 assays to assess drug transport and the potential for drug–drug interaction to support regulatory submissions. *Xenobiotica*.

[17] Eng H., Scialis R. J., Rotter C. J. (2016). The antimicrobial agent fusidic acid inhibits organic anion transporting polypeptide-mediated hepatic clearance and may potentiate statin-induced myopathy. *Drug Metabolism and Disposition*.

[18] Gupta A., Harris J. J., Lin J., Bulgarelli J. P., Birmingham B. K., Grimm S. W. (2016). Fusidic acid inhibits hepatic transporters and metabolic enzymes: potential cause of clinical drug-drug interaction observed with statin coadministration. *Antimicrobial Agents and Chemotherapy*.

[19] Boyd G. S., Merrick M. V., Monks R., Thomas I. L. (1981). Se-75-labeled bile acid analogs, new radiopharmaceuticals for investigating the enterohepatic circulation. *Journal of Nuclear Medicine*.

[20] Schmassmann A., Fehr H. F., Locher J. (1993). Cholylsarcosine, a new bile acid analogue: metabolism and effect on biliary secretion in humans. *Gastroenterology*.

[21] Frisch K., Jakobsen S., Sorensen M. (2012). N-methyl-11C]cholylsarcosine, a novel bile acid tracer for PET/CT of hepatic excretory function: radiosynthesis and proof-of-concept studies in pigs. *Journal of Nuclear Medicine*.

[22] Orntoft N. W., Munk O. L., Frisch K., Ott P., Keiding S., Sorensen M. (2017). Hepatobiliary transport kinetics of the conjugated bile acid tracer 11C-CSar quantified in healthy humans and patients by positron emission tomography. *Journal of Hepatology*.

[23] De Lombaerde S., Neyt S., Kersemans K. (2017). Synthesis, in vitro and in vivo evaluation of 3beta-[18F]fluorocholic acid for the detection of drug-induced cholestasis in mice. *PLoS One*.

[24] Konig J., Klatt S., Dilger K., Fromm M. F. (2012). Characterization of ursodeoxycholic and norursodeoxycholic acid as substrates of the hepatic uptake transporters OATP1B1, OATP1B3, OATP2B1 and NTCP. *Basic & Clinical Pharmacology & Toxicology*.

[25] Stieger B. (2011). The role of the sodium-taurocholate cotransporting polypeptide (NTCP) and of the bile salt export pump (BSEP) in physiology and pathophysiology of bile formation. *Handbook of Experimental Pharmacology*.

[26] Kullak-Ublick G. A., Stieger B., Hagenbuch B., Meier P. J. (2000). Hepatic transport of bile salts. *Seminars in Liver Disease*.

[27] Sorensen M., Munk O. L., Orntoft N. W. (2016). Hepatobiliary secretion kinetics of conjugated bile acids measured in pigs by 11C-cholylsarcosine PET. *Journal of Nuclear Medicine*.

[28] Mita S., Suzuki H., Akita H. (2006). Inhibition of bile acid transport across Na+/taurocholate cotransporting polypeptide (SLC10A1) and bile salt export pump (ABCB 11)-coexpressing LLC-PK1 cells by cholestasis-inducing drugs. *Drug Metabolism and Disposition*.

[29] Stieger B., Fattinger K., Madon J., Kullak-Ublick G. A., Meier P. J. (2000). Drug- and estrogen-induced cholestasis through inhibition of the hepatocellular bile salt export pump (BSEP) of rat liver. *Gastroenterology*.

[30] Wang E. J., Casciano C. N., Clement R. P., Johnson W. W. (2003). Fluorescent substrates of sister-P-glycoprotein (BSEP) evaluated as markers of active transport and inhibition: evidence for contingent unequal binding sites. *Pharmaceutical Research*.

[31] Dawson S., Stahl S., Paul N., Barber J., Kenna J. G. (2012). In vitro inhibition of the bile salt export pump correlates with risk of cholestatic drug-induced liver injury in humans. *Drug Metabolism and Disposition*.

[32] Neyt S., Huisman M. T., Vanhove C. (2013). In vivo visualization and quantification of (Disturbed) Oatp-mediated hepatic uptake and Mrp2-mediated biliary excretion of 99mTc-mebrofenin in mice. *Journal of Nuclear Medicine*.

[33] Lapham K., Novak J., Marroquin L. D. (2016). Inhibition of hepatobiliary transport activity by the antibacterial agent fusidic acid: insights into factors contributing to conjugated hyperbilirubinemia/cholestasis. *Chemical Research in Toxicology*.

[34] Akita H., Suzuki H., Ito K. (2001). Characterization of bile acid transport mediated by multidrug resistance associated protein 2 and bile salt export pump. *Biochimica et Biophysica Acta*.

[35] Bode K. A., Donner M. G., Leier I., Keppler D. (2002). Inhibition of transport across the hepatocyte canalicular membrane by the antibiotic fusidate. *Biochemical Pharmacology*.

