# Supplementary material for: Preclinical Evaluation of [18F]LCATD as a PET Tracer to Study Drug-Drug Interactions Caused by Inhibition of Hepatic Transporters
Source: Contrast Media Mol Imaging. 2018 Jul 30;2018:3064751. doi: 10.1155/2018/3064751 (PMC6091370; doi:10.1155/2018/3064751)
Supplement: Supplementary Materials — The supporting information includes radio-HPLC analysis of bile extracts (Figure S1); the experimentalprotocol for the radiosynthesis of [ 18 F]LCATD,module configuration (.doc file) (Figure S2); HPLC coinjectionwith LCATD (Figure S3); and visualisation of ROIs (Figure S4). Rotating ROIs are visualised in the movie file Video_ROIs.wmv. [file 3064751.f1.zip › Supporting Information_CMMI_2306298.docx]

**SUPPORTING INFORMATION**

**Graphical Abstract**


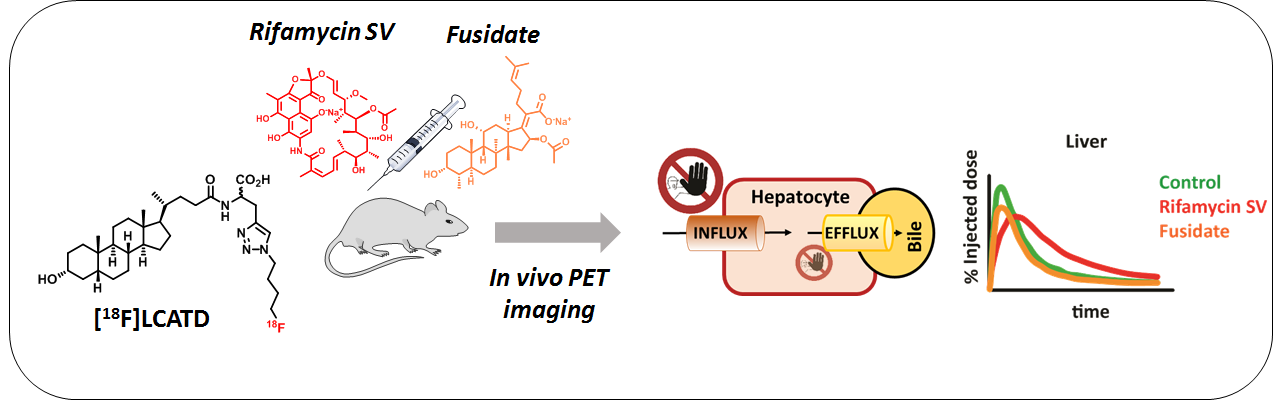


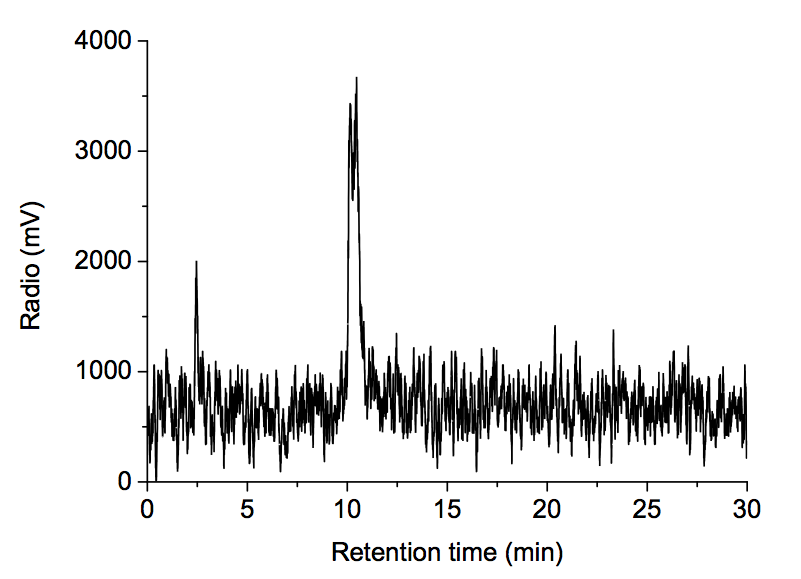


**Figure S1.**Radio- HPLC profile of the bile extract of a representative control rat. The peaks at 10.08 minutes correspond to the two [^18^F]LCATD diastereoisomers.

**Radiosynthesis of [^18^F]LCATD**

Radiosynthesis, purification and formulation steps were performed in an Eckert & Ziegler ModularLab system equipped with semipreparative HPLC. [^18^F]Fluoride (∼16 GBq) was delivered from the cyclotron into a stock vial and then separated from the [^18^O]H_2_O by means of a Chromafix PS-HCO3-ion exchange cartridge (Waters). [^18^F]Fluoride was then eluted with the solution of K_2_HPO_4_ (5 mg in 0.5 mL of sterile water) and collected in the reactor, followed by the solution of Kryptofix K222 (15 mg in 1 mL of anhydrous CH_3_CN). A first drying cycle was accomplished by heating at 95 °C under both vacuum and helium flow (400 s) and then under vacuum only (150 s) before delivering an additional amount of anhydrous CH_3_CN. The drying process was repeated and the solution of mesylate precursor (prepared according to *Bioorg. Med. Chem.* **2017**, *25*, 963-976) (5 mg in 1 mL of anhydrous CH_3_CN) was then delivered into the reactor containing the [^18^F]KF-K222 dry complex. The mixture was heated at 90 °C for 15 min. The reactor was cooled to 40 °C before adding the sodium hydroxyde solution (0.5 mL, 2 M in 50% aqueous methanol) which was allowed to react for 10 min. After the hydrolysis step, the crude reaction mixture was neutralized with 2M aqueous HCl (0.5 mL) and pumped into a vial containing the HPLC eluent (PBS/CH_3_CN 60:40, 1.5 mL). The mixture (about 3 mL) was then loaded in the 5 mL RP-HPLC loop and then injected in the Phenomenex Luna C18 column (5 μm, 250 × 10 mm, 100 Å), which was eluted with PBS/CH_3_CN 60:40 (5 mL min-1 flow). The peak corresponding to the desired product was collected in a vial containing 50 mL of distilled water. The diluted solution of pure [^18^F]LCATD was then flushed through a C18 SPE cartridge Oasis® HLB Plus (Waters) in order to trap the tracer. The cartridge was then eluted with absolute ethanol (0.8 mL) and the resulting [^18^F]LCATD was passed through a sterile filter and finally delivered into a vial, placed in a second hot cell, containing 7.2 mL of sterile PBS. Up to 2 GBq of injectable solution of [^18^F]LCATD were obtained (8 mL, up to 250 MBq mL^−1^). The total synthesis, purification and formulation time was about 90 min. The tracer was obtained in 25 ± 5% (n = 5) decay corrected radiochemical yield. HPLC analysis of the formulated product confirmed the identity (co-injection with cold reference material) and the purity of the tracer (RCP > 99%).


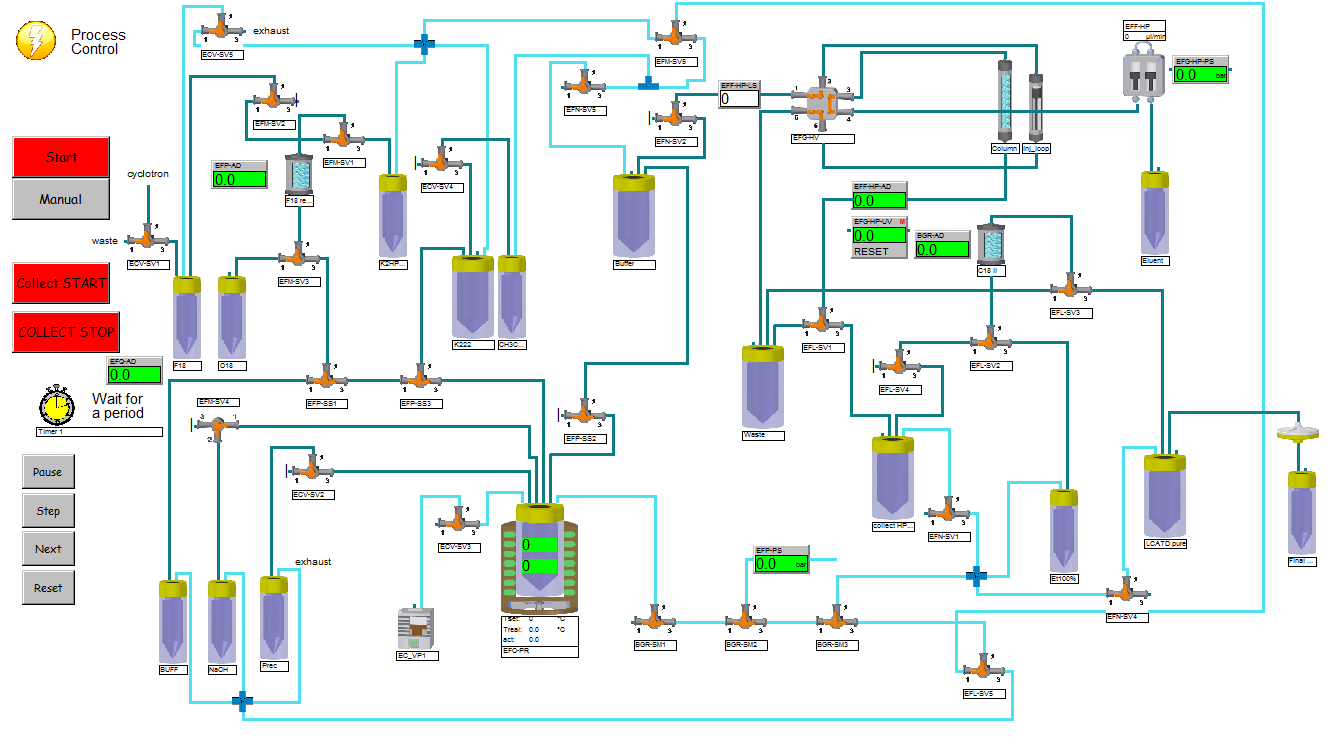


**Figure S2. Configuration of Eckert & Ziegler module for production of [^18^F]LCATD**

Co-injection of formulated [^18^F]**LACTD** and cold **LCATD** reference compound.


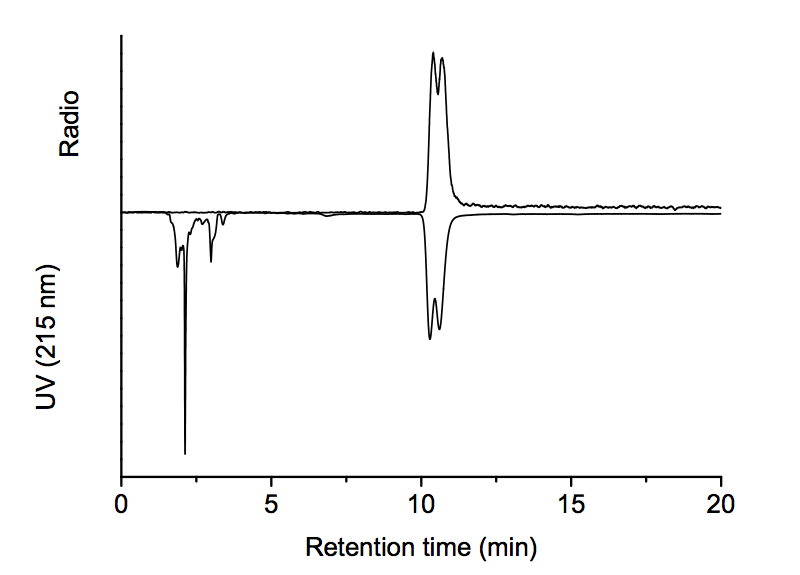


**Figure S3.** Co-injection of formulated [^18^F]LCATD and cold LCATD reference (t_R_ = 10.35 and 10.66 min).

**
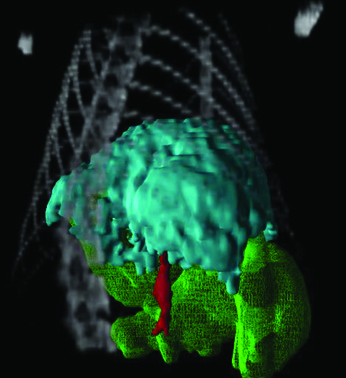
**

**FIGURE S4:** ROIs for the quantification of the activity in the blood (abdominal aorta, in red), liver (in cyan), and bile (gastrointestinal tract, green).

**Supplementary movie (see the attached file).** Rotating view of ROIs for the quantification of the activity in the blood (abdominal aorta, in red), liver (in cyan), and bile (gastrointestinal tract, green).
