# Supplementary material for: Preclinical Evaluation of [18F]LCATD as a PET Tracer to Study Drug-Drug Interactions Caused by Inhibition of Hepatic Transporters
Source: Contrast Media Mol Imaging. 2018 Jul 30;2018:3064751. doi: 10.1155/2018/3064751 (PMC6091370; doi:10.1155/2018/3064751)
Supplement: Supplementary Materials — The supporting information includes radio-HPLC analysis of bile extracts (Figure S1); the experimentalprotocol for the radiosynthesis of [ 18 F]LCATD,module configuration (.doc file) (Figure S2); HPLC coinjectionwith LCATD (Figure S3); and visualisation of ROIs (Figure S4). Rotating ROIs are visualised in the movie file Video_ROIs.wmv. [file 3064751.f1.zip › Supporting Information_CMMI_2306299.pdf]

SUPPORTING INFORMATION

Graphical Abstract

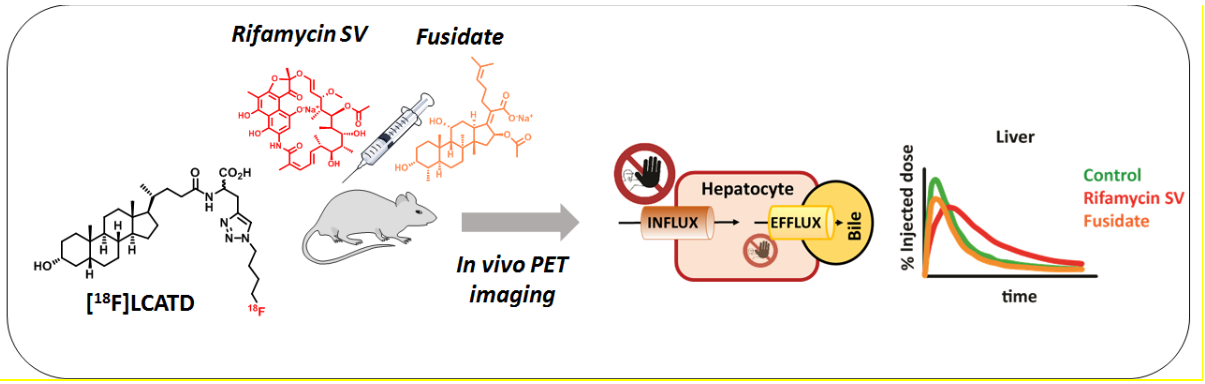

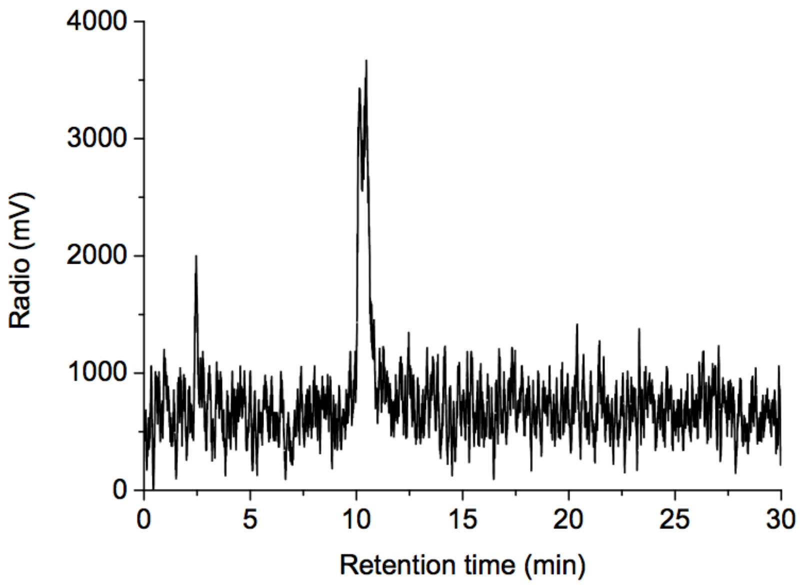

Figure S1. Radio- HPLC profile of the bile extract of a representative control rat. The peaks at 10.08 minutes correspond to the two [^{18}F]LCATD diastereoisomers.

Radiosynthesis of [^{18}F]LCATD

Radiosynthesis, purification and formulation steps were performed in an Eckert & Ziegler ModularLab system equipped with semipreparative HPLC. [^{18}F]Fluoride (~16 GBq) was delivered from the cyclotron into a stock vial and then separated from the [^{18}O]H_2O by means of a Chromafix PS-HCO3-ion exchange cartridge (Waters). [^{18}F]Fluoride was then eluted with the solution of K_2HPO_4 (5 mg in 0.5 mL of sterile water) and collected in the reactor, followed by the solution of Kryptofix K222 (15 mg in 1 mL of anhydrous CH_3CN). A first drying cycle was accomplished by heating at 95 °C under both vacuum and helium flow (400 s) and then under vacuum only (150 s) before delivering an additional amount of anhydrous CH_3CN. The drying process was repeated and the solution of mesylate precursor (prepared according to *Bioorg. Med. Chem.* **2017**, *25*, 963-976)

(5 mg in 1 mL of anhydrous CH_3CN) was then delivered into the reactor containing the [^{18}F]KF-K222 dry complex. The mixture was heated at 90 °C for 15 min. The reactor was cooled to 40 °C before adding the sodium hydroxyde solution (0.5 mL, 2 M in 50% aqueous methanol) which was allowed to react for 10 min. After the hydrolysis step, the crude reaction mixture was neutralized with 2M aqueous HCl (0.5 mL) and pumped into a vial containing the HPLC eluent (PBS/CH_3CN 60:40, 1.5 mL). The mixture (about 3 mL) was then loaded in the 5 mL RP-HPLC loop and then injected in the Phenomenex Luna C18 column (5 µm, 250 × 10 mm, 100 Å), which was eluted with PBS/CH_3CN 60:40 (5 mL min-1 flow). The peak corresponding to the desired product was collected in a vial containing 50 mL of distilled water. The diluted solution of pure [^{18}F]LCATD was then flushed through a C18 SPE cartridge Oasis® HLB Plus (Waters) in order to trap the tracer. The cartridge was then eluted with absolute ethanol (0.8 mL) and the resulting [^{18}F]LCATD was passed through a sterile filter and finally delivered into a vial, placed in a second hot cell, containing 7.2 mL of sterile PBS. Up to 2 GBq of injectable solution of [^{18}F]LCATD were obtained (8 mL, up to 250 MBq mL^{-1}). The total synthesis, purification and formulation time was about 90 min. The tracer was obtained in 25 ± 5% (n = 5) decay corrected radiochemical yield. HPLC analysis of the formulated product confirmed the identity (co-injection with cold reference material) and the purity of the tracer (RCP > 99%).

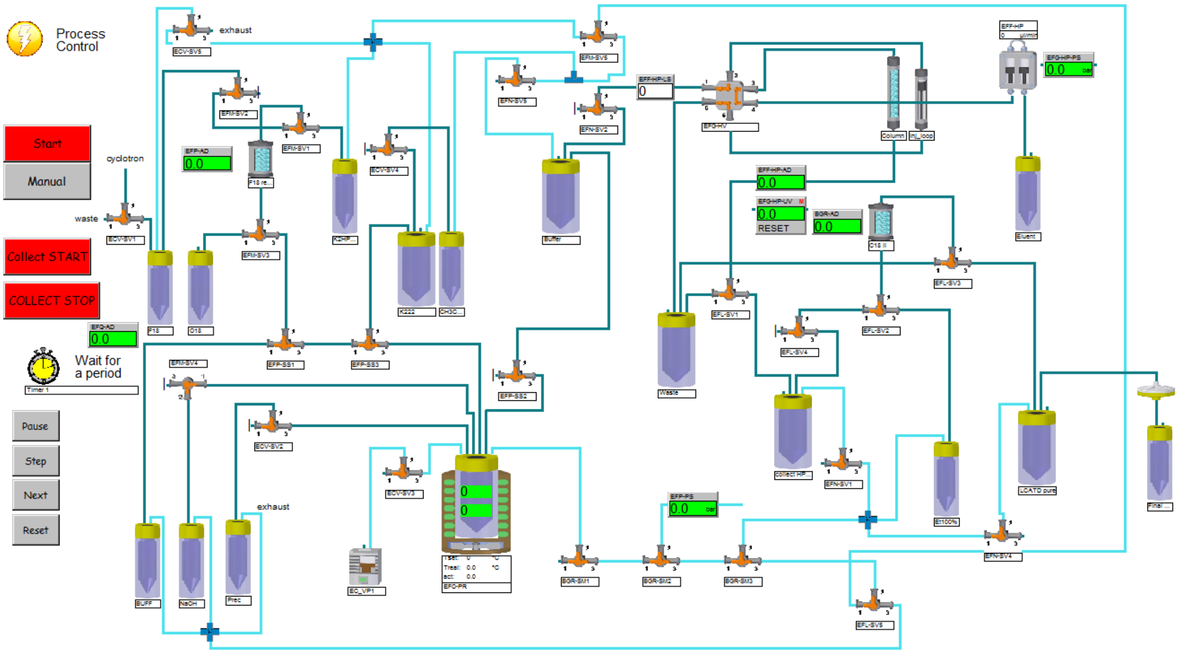

Figure S2. Configuration of Eckert & Ziegler module for production of [^{18}F]LCATD

Co-injection of formulated [^{18}F]**LACTD** and cold **LCATD** reference compound.

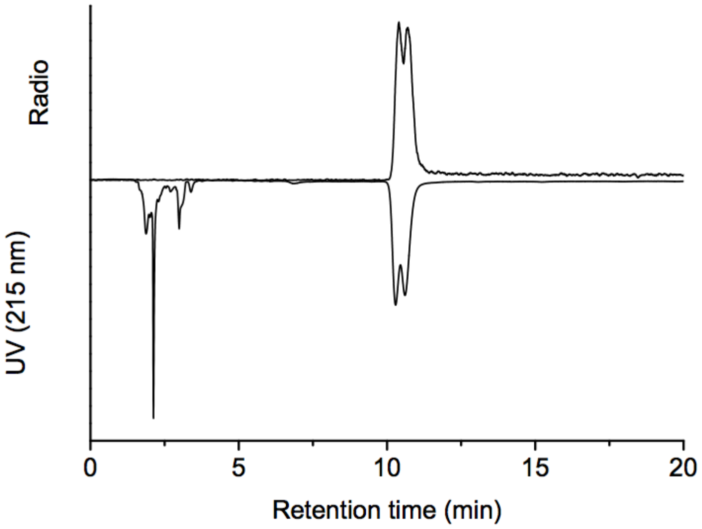

Figure S3. Co-injection of formulated [^{18}F]LCATD and cold LCATD reference (t_R = 10.35 and 10.66 min).

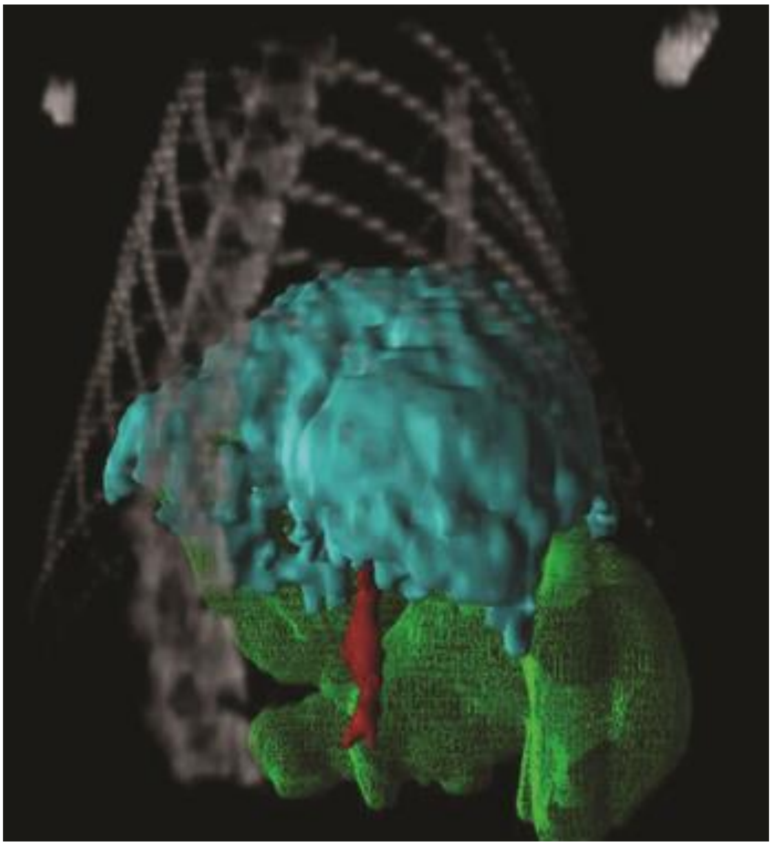

FIGURE S4: ROIs for the quantification of the activity in the blood (abdominal aorta, in red), liver (in cyan), and bile (gastrointestinal tract, green).

Supplementary movie (see the attached file). Rotating view of ROIs for the quantification of the activity in the blood (abdominal aorta, in red), liver (in cyan), and bile (gastrointestinal tract, green).
