# Supplementary figures and images for: Preclinical Evaluation of [18F]LCATD as a PET Tracer to Study Drug-Drug Interactions Caused by Inhibition of Hepatic Transporters
Source: Contrast Media Mol Imaging. 2018 Jul 30;2018:3064751. doi: 10.1155/2018/3064751 (PMC6091370; doi:10.1155/2018/3064751)

## Slide 1
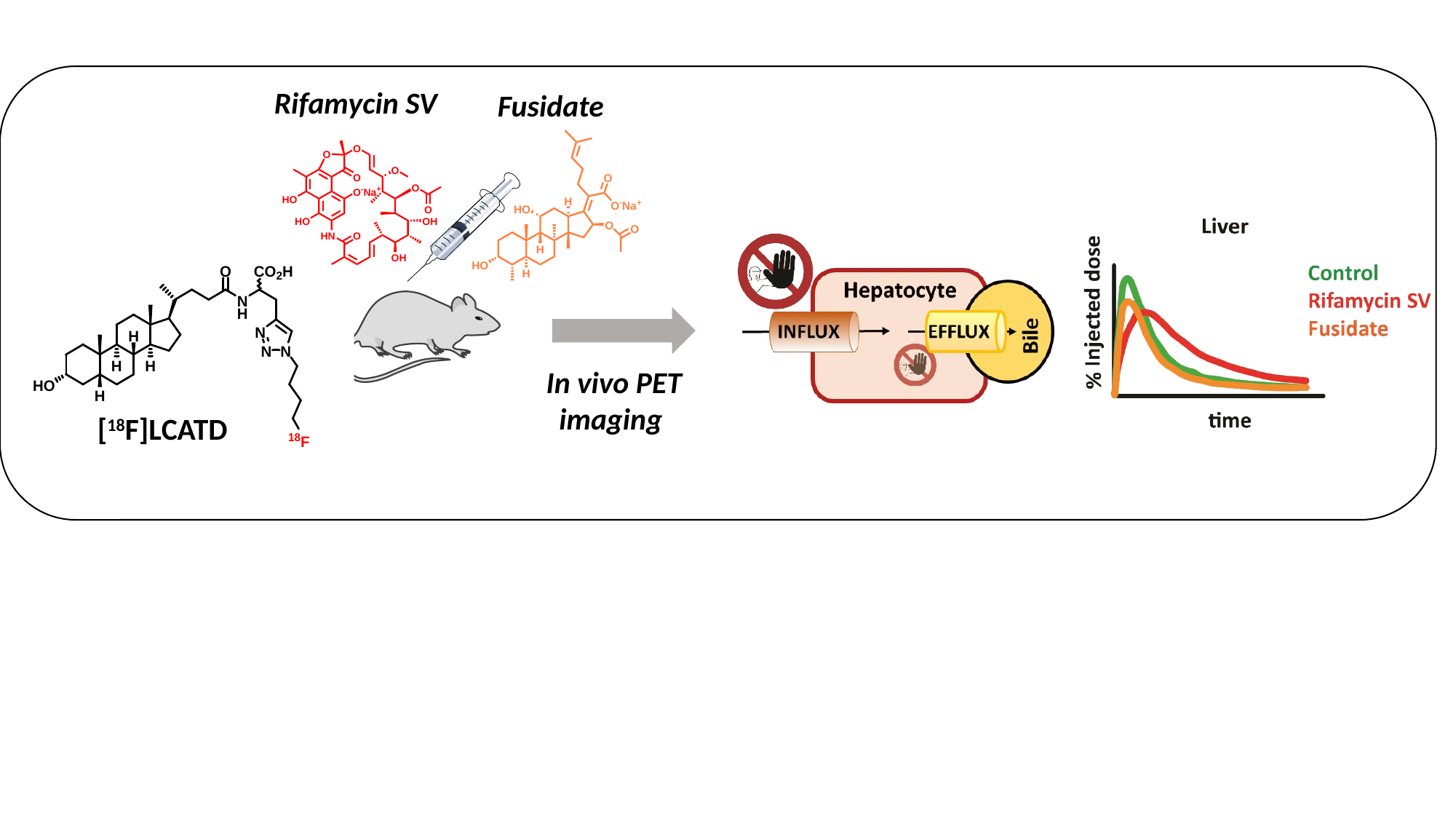

Rifamycin SV
Fusidate
In vivo PET imaging
[18F]LCATD

Supplement: Supplementary Materials — The supporting information includes radio-HPLC analysis of bile extracts (Figure S1); the experimentalprotocol for the radiosynthesis of [ 18 F]LCATD,module configuration (.doc file) (Figure S2); HPLC coinjectionwith LCATD (Figure S3); and visualisation of ROIs (Figure S4). Rotating ROIs are visualised in the movie file Video_ROIs.wmv. [file 3064751.f1.zip › GA_REVISED_CMMI_2306295.pptx]
